# NF-κB Signaling as a Central Driver of Cancer Cachexia

**DOI:** 10.3390/cancers18040557

**Published:** 2026-02-09

**Authors:** Yan Li, Hao Jiang, Rui Chen, Haitao Huang, Shengguang Ding

**Affiliations:** 1 Department of Cardiovascular Surgery, Southeast University Affiliated Nantong First People’s Hospital, Nantong 226001, China; 2113320228@stmail.ntu.edu.cn; 2 School of Medicine, Nantong University, Nantong 226001, China; jianghaohmqzyy@163.com; 3 Department of General Surgery, Haimen Traditional Chinese Medicine Hospital, Haimen 226100, China; 4 Department of Thoracic Surgery, Southeast University Affiliated Nantong First People’s Hospital, Nantong 226001, China; 5 Department of Pathology, Medical School of Nantong University, Nantong 226001, China; 2331310038@stmail.ntu.edu.cn

**Keywords:** NF-κB signaling, cancer cachexia, muscle wasting, adipose tissue remodeling, systemic inflammation, therapeutic targeting

## Abstract

Cancer cachexia is a multifactorial metabolic syndrome characterized by progressive skeletal muscle wasting, chronic systemic inflammation, and profound metabolic imbalance. Sustained activation of the nuclear factor κB (NF-κB) signaling pathway lies at the core of its pathogenesis, driving muscle proteolysis, impairing regenerative capacity, disrupting adipose tissue homeostasis, and promoting insulin resistance and anorexia. By transcriptionally regulating catabolic and pro-inflammatory gene programs across skeletal muscle, adipose tissue, the liver, and the central nervous system, NF-κB establishes a self-amplifying inflammatory–metabolic loop that perpetuates tissue wasting and systemic dysfunction. Accumulating preclinical and clinical evidence identifies NF-κB as a viable therapeutic target in cancer cachexia. Pharmacologic inhibitors (e.g., SR12343, DHMEQ), anti-inflammatory strategies (e.g., nonsteroidal anti-inflammatory drugs and IL-6 receptor–targeting antibodies), and nutritional interventions (e.g., omega-3 fatty acids) have shown efficacy in attenuating cachexia-associated inflammation, metabolic dysregulation, and tissue loss. Notably, emerging multimodal approaches integrating NF-κB modulation with metabolic support, chemotherapy, and behavioral interventions demonstrate synergistic benefits. This review integrates current mechanistic insights and therapeutic advances, highlighting NF-κB as a central pathogenic axis and a compelling target for translational intervention in cancer cachexia.

## Simple Summary

Cancer cachexia is a debilitating complication that profoundly affects patient quality of life and treatment outcomes, yet its underlying mechanisms remain incompletely understood. Accumulating evidence indicates that persistent inflammation plays a central role in driving muscle wasting, adipose tissue remodeling, and systemic metabolic imbalance. This article provides an integrated overview of how inflammatory signaling coordinates pathological changes across multiple organs, highlighting its contribution to the progression of cachexia. By synthesizing current mechanistic insights, this work aims to clarify key regulatory pathways and identify unifying concepts that advance understanding of disease pathogenesis. These insights may help guide future research and support the development of more effective, mechanism-based therapeutic strategies for cancer cachexia.

## 1. Introduction

Cancer cachexia is a multifactorial metabolic syndrome characterized by progressive skeletal muscle wasting, unintentional weight loss, and profound systemic energy imbalance. Importantly, cancer cachexia is more than malnutrition; it represents a hypermetabolic, paraneoplastic syndrome that is largely unresponsive to conventional nutritional support. It is driven by chronic inflammation, enhanced proteolysis, and impaired tissue regeneration, and it is strongly associated with poor treatment tolerance, reduced quality of life, and increased mortality [1,2]. In addition to muscle wasting, cancer cachexia is characterized by maladapted adipose tissue metabolism, including loss of white adipose tissue and pathological browning, together with systemic inflammation marked by elevated TNF-α, IL-6, IL-1β, and related cytokines. Among the numerous molecular drivers implicated in cachexia, the nuclear factor κB (NF-κB) signaling pathway has emerged as a central orchestrator of disease pathophysiology [3]. These features collectively define a cachectic cascade in which inflammatory and metabolic alterations reinforce one another. Pro-inflammatory cytokines such as tumor necrosis factor-α (TNF-α), interleukin-6 (IL-6), and interleukin-1β (IL-1β), secreted by tumors and immune cells, activate the IκB kinase (IKK) complex, leading to phosphorylation and degradation of IκB inhibitors. [4,5]. This process permits nuclear translocation of NF-κB dimers predominantly p50/RelA—which induce transcription of key catabolic genes, including *MuRF1*, *Atrogin-1*, and *iNOS* [3]. Consequently, both the ubiquitin–proteasome system (UPS) and the autophagy–lysosome pathway (ALP) are activated, accelerating myofibrillar protein degradation [6]. In parallel, TNF-α suppresses the IGF-1/Akt/mTOR anabolic signaling cascade, further exacerbating the imbalance between protein synthesis and degradation [7,8]. Together, these processes position NF-κB as a critical molecular link between inflammation and muscle atrophy in cancer cachexia.

Beyond its role in proteolysis, sustained NF-κB activation profoundly disrupts muscle regeneration and tissue homeostasis. NF-κB upregulates Pax7 in satellite cells while repressing myogenic regulators such as *Cyclin D1* and *MyoD*, thereby impairing myoblast differentiation and regeneration of damaged fibers [9]. Importantly, NF-κB signaling integrates multiple components of the cachectic cascade across different cellular compartments. NF-κB activation occurs across multiple cellular compartments including myofibers, fibro-adipogenic progenitors (FAPs), and infiltrating macrophages forming a multicellular inflammatory feedback loop that perpetuates local tissue degeneration [3]. Elevated reactive oxygen species (ROS) further amplify NF-κB signaling and cooperate with FOXO transcription factors to reinforce UPS and ALP mediated proteolysis while suppressing mTOR activity [10]. In addition, cytokine crosstalk particularly between IFN-γ and TNF-α engages the STAT3–NF-κB axis, promoting *iNOS* expression and intensifying catabolic signaling [11]. Collectively, this interconnected network of inflammation, oxidative stress, and regenerative failure underscores the pivotal role of NF-κB in skeletal muscle dysfunction during cachexia [12,13].

Mechanistically, NF-κB is a highly conserved intracellular signaling pathway that translates diverse extracellular cues including cytokines, pathogen-associated ligands, and oxidative stress into specific gene expression programs. As a central node of the cachexia cascade, NF-κB links inflammatory signaling to tissue-specific metabolic remodeling. The NF-κB family comprises five subunits (p50, p52, RelA/p65, RelB, and c-Rel), all of which share a Rel homology domain responsible for DNA binding and dimerization [14,15]. Canonical NF-κB signaling is activated by stimuli such as TNF-α, IL-1β, and Toll-like receptor ligands through TAK1-mediated phosphorylation of IKKβ within the IKK complex (IKKα, IKKβ, and NEMO/IKKγ), leading to IκBα degradation and nuclear translocation of NF-κB dimers [16]. In contrast, the non-canonical pathway relies on NF-κB-inducing kinase (NIK) and IKKα to promote p100 processing into p52 and nuclear accumulation of RelB-containing dimers, primarily regulating adaptive immune responses [17,18]. While transient NF-κB activation is essential for immune defense and tissue repair, chronic or dysregulated activation drives persistent inflammation, progressive muscle wasting, adipose tissue remodeling, and tumor progression [19]. Consistent with this, preclinical studies demonstrate that both genetic and pharmacological inhibition of NF-κB using IKK inhibitors (e.g., DHMEQ, SR12343), natural compounds such as curcumin, or low-dose nonsteroidal anti-inflammatory drugs (NSAIDs) can attenuate muscle wasting and preserve lean mass [20,21,22]. These findings establish NF-κB as a central molecular node in cancer cachexia and support its therapeutic targeting through multimodal strategies that integrate anti-inflammatory agents, metabolic modulation, and exercise-based rehabilitation. Accordingly, this review aims to delineate the multifaceted roles of NF-κB in cancer cachexia across skeletal muscle, adipose tissue, systemic metabolism, and central appetite regulation, and to discuss current and emerging therapeutic strategies targeting this pathway.

## 2. Overview of NF-κB Signaling Pathway

The NF-κB family comprises five evolutionarily conserved transcription factors p50 (processed from p105), p52 (from p100), RelA (p65), RelB, and c-Rel each containing a Rel Homology Domain (RHD) at the N-terminus that facilitates DNA binding, dimerization, and interaction with inhibitory IκB proteins [23,24,25]. Under basal conditions, NF-κB dimers are retained through association with IκB family members or precursor proteins containing ankyrin repeats. [26,27]. NF-κB signaling is classically divided into two major branches: the canonical and non-canonical pathways, which differ in activation triggers, signaling kinetics, and biological outputs [15,28].

In the canonical pathway, pro-inflammatory stimuli such as TNF-α, IL-1β, Toll-like receptor ligands, and chemotherapy-associated stress activate transforming growth factor-β–activated kinase 1 (TAK1), leading to phosphorylation of the IKK complex [28,29]. IKKβ-mediated degradation of IκBα permits rapid nuclear translocation of RelA/p50 heterodimers, inducing transcription of genes involved in inflammation, stress responses, and cellular metabolism [30]. Additionally, IKKβ phosphorylates p65 on Ser536, enhancing its transcriptional potency [31]. Functionally, this branch predominates in acute inflammatory responses and stress responses and, under conditions of sustained activation, contributes to pathological processes such as skeletal muscle atrophy in cancer cachexia through induction of MuRF1, Atrogin-1, and *iNOS* [3,9,32,33]. In contrast, the non-canonical NF-κB pathway is selectively activated by a restricted subset of tumor necrosis factor receptor superfamily members, including CD40, BAFF-R, and lymphotoxin-β receptor. This pathway depends on stabilization of NF-κB-inducing kinase (NIK) and IKKα-mediated processing of p100 into p52, generating p52/RelB heterodimers that regulate genes involved primarily in immune cell differentiation and lymphoid homeostasis [15,34]. Compared with canonical signaling, non-canonical activation is slower and relies on de novo protein synthesis. Importantly, current evidence supporting a dominant role for NF-κB signaling in cancer cachexia is derived predominantly from experimental models in which the canonical IKKβ–RelA axis is persistently activated. In widely used cachexia models including C26 colon carcinoma, Lewis lung carcinoma, and Apc^Min/+ mice canonical NF-κB signaling drives skeletal muscle proteolysis, adipose tissue remodeling, and systemic inflammation. In contrast, the contribution of non-canonical NF-κB signaling to cachexia appears to be context-dependent and remains incompletely defined, with available data suggesting a more indirect role in immune remodeling rather than direct induction of tissue wasting.

**Figure 1:**
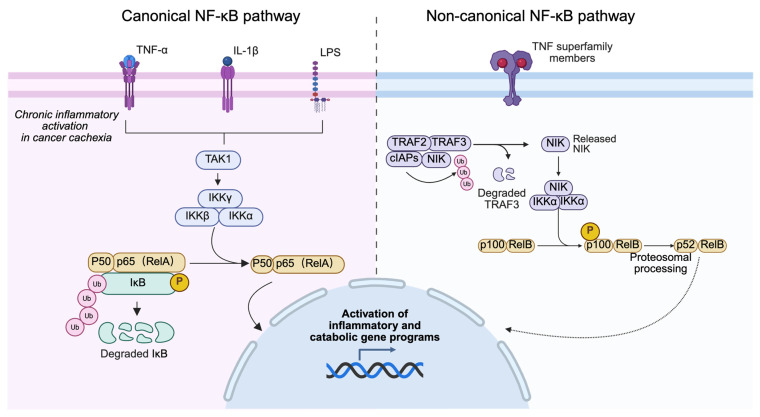
Canonical and non-canonical NF-κB signaling pathways in cancer cachexia. NF-κB signaling operates through two major branches, the canonical and non-canonical pathways, which differ in their upstream triggers, signaling dynamics, and biological functions. In the canonical pathway, pro-inflammatory stimuli including TNF-α, IL-1β, and LPS activate TAK1, leading to activation of the IKK complex. Phosphorylation and subsequent proteasomal degradation of IκB proteins release p50/p65 (RelA) heterodimers, enabling their nuclear translocation and transcriptional activation. In experimental models of cancer cachexia, sustained activation of the canonical NF-κB pathway rather than transient physiological signaling drives chronic inflammation and induction of pro-inflammatory and catabolic gene programs associated with tissue wasting. By contrast, the non-canonical pathway is selectively triggered by specific tumor necrosis factor receptor superfamily members through NF-κB-inducing kinase (NIK) stabilization and IKKα-dependent processing of p100 into p52, generating p52/RelB heterodimers that primarily regulate immune homeostasis. Collectively, current evidence derived predominantly from preclinical cachexia models supports a predominant role for canonical NF-κB signaling in inflammation-associated metabolic dysfunction, while the contribution of non-canonical signaling appears to be context-dependent and less well defined.

Despite their distinct upstream regulators, canonical and non-canonical pathways are interconnected through regulatory crosstalk that fine-tunes NF-κB activity [28,35]. However, in the context of cancer cachexia, sustained activation of the canonical pathway rather than balanced physiological signaling emerges as the principal mechanism linking chronic inflammation to metabolic and catabolic dysfunction [22]. Accordingly, subsequent sections focus on cachexia-specific NF-κB activation patterns and downstream pathological consequences across target tissues (Figure 1).

## 3. NF-κB Activation via Pro-Inflammatory Cytokines: Linking Inflammation to Muscle Wasting

### 3.1. Cytokine-Driven NF-κB Activation and Its Catabolic Impact in Cancer Cachexia

Cancer cachexia is characterized by chronic systemic inflammation, with elevated circulating levels of TNF-α, IL-1β, IL-6, and interferon-γ (IFN-γ) [36]. These cytokines converge on NF-κB signaling in skeletal muscle, establishing a pro-catabolic transcriptional program that disrupts muscle proteostasis [3]. In experimental cachexia models, TNF-α robustly activates the canonical IKKβ–NF-κB pathway, leading to upregulation of muscle-specific E3 ubiquitin ligases such as *MuRF1* and *Atrogin-1*, thereby accelerating ubiquitin–proteasome-mediated protein degradation [37,38]. IL-1β further amplifies muscle catabolism through NF-κB-dependent induction of cyclooxygenase-2 and hypoxia-responsive pathways, while IL-6 activates the gp130/JAK/STAT3 axis and synergistically enhances NF-κB signaling [39,40,41]. IFN-γ cooperates with TNF-α to promote STAT3–NF-κB complex formation, driving *iNOS* expression and oxidative stress–associated proteolysis [42,43]. Together, these cytokines generate a feed-forward inflammatory loop that sustains NF-κB activation and progressive muscle wasting [14]. Notably, the majority of mechanistic evidence supporting these pathways originates from preclinical models, where pharmacologic or genetic inhibition of IKKβ or RelA consistently attenuates muscle loss. In contrast, human studies largely demonstrate correlations between circulating inflammatory cytokines, NF-κB target gene expression, and cachexia severity, underscoring a gap between mechanistic validation and clinical causality.

### 3.2. NF-κB as a Central Integrator of Systemic Inflammation in Cachexia

In cancer cachexia, NF-κB functions as a context dependent integrator of chronic inflammatory signals rather than a generic or uniformly acting inflammatory switch. At the core of these inflammatory responses lies NF-κB, a master transcriptional regulator that integrates extracellular inflammatory cues into gene expression programs governing immunity, metabolism, and tissue homeostasis [28,44]. In unstimulated cells, NF-κB dimers (primarily p65/p50) are sequestered in the cytoplasm by inhibitory IκB proteins [45]. Upon activation of Toll-like receptors (TLRs) or cytokine receptors (e.g., TNFR, IL-1R), the IKK complex phosphorylates IκB, marking it for proteasomal degradation and releasing NF-κB to translocate into the nucleus [46]. Once activated, NF-κB drives the expression of pro-inflammatory cytokines, chemokines, and adhesion molecules, thereby amplifying inflammatory cascades [47,48]. Importantly, the pathological relevance of NF-κB signaling in cancer cachexia arises from its sustained and cell type specific activation across multiple tissues. In macrophages, NF-κB upregulates TNF-α and IL-6, reinforcing systemic inflammation; in endothelial cells, it promotes leukocyte recruitment through induction of VCAM-1 and ICAM-1; and in adipocytes, TLR4 activation by free fatty acids engages NF-κB signaling, contributing to metabolic stress and insulin resistance [49,50,51]. A key regulatory feature of NF-κB is its self-reinforcing loop TNF-α induces NF-κB, which in turn promotes further TNF-α transcription, sustaining inflammation [52]. This persistent activation state enables NF-κB to integrate systemic inflammatory cues into coordinated transcriptional programs that promote tissue catabolism and metabolic dysregulation. Although targeting NF-κB signaling (e.g., via IKKβ inhibitors or p65 silencing) shows clear efficacy in experimental models, therapeutic translation remains challenging due to the risk of impairing essential immune and regenerative functions [53].

### 3.3. Synergistic Interactions Between NF-κB, STAT3, and p38 MAPK Signaling

**Figure 2:**
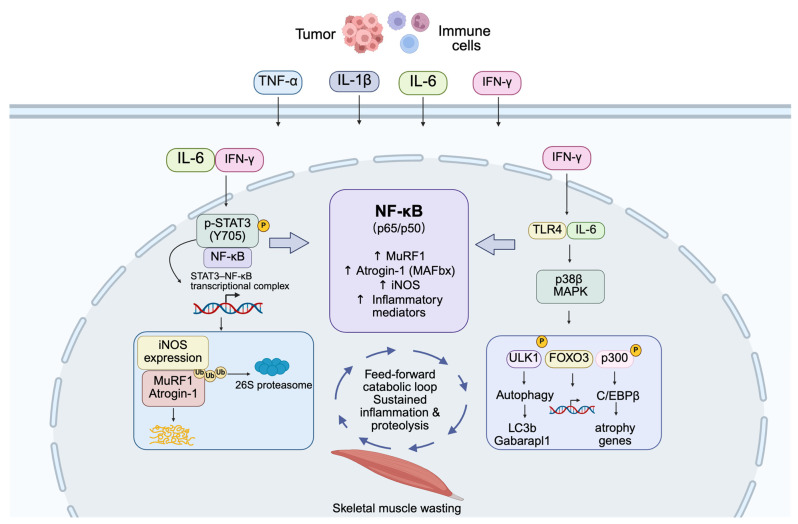
NF-κB-centered inflammatory signaling network driving skeletal muscle wasting in cancer cachexia. Tumor- and immune cell-derived pro-inflammatory cytokines, including TNF-α, IL-1β, IL-6, and IFN-γ, converge on skeletal muscle fibers to activate inflammatory signaling pathways. These cues promote nuclear factor κB (NF-κB; p65/p50) activation, which acts as a key coordinating transcriptional node integrating inflammatory and stress-responsive signals in cachectic skeletal muscle. NF-κB induces the expression of muscle-specific E3 ubiquitin ligases MuRF1 and Atrogin-1 (MAFbx), *iNOS*, and pro-inflammatory mediators, thereby activating the ubiquitin–proteasome system. In parallel, IL-6- and IFN-γ-dependent phosphorylation of STAT3 (Y705) enables the formation of STAT3–NF-κB transcriptional complexes that further amplify catabolic gene expression. Concurrently, TLR4 and IL-6 signaling activate p38β MAPK, which promotes autophagy through ULK1 and LC3b/Gabarapl1 and induces atrophy related gene programs via FOXO3 and p300/C/EBPβ. Together, these interconnected pathways establish a feed-forward catabolic network in experimental models of cancer cachexia, sustaining inflammation, proteolysis, and progressive skeletal muscle wasting.

In cachectic skeletal muscle, NF-κB activation cooperates with other catabolic pathways to exacerbate protein degradation [8,54]. One prominent axis involves STAT3, which is activated by IL-6, IFN-γ, and TNF-α. Upon phosphorylation (Y705), STAT3 forms a complex with NF-κB (p65), and together they translocate to the nucleus to functionally cooperate with NF-κB to enhance *iNOS/NO* catabolic signaling, triggering the *iNOS/NO* catabolic cascade [42]. Persistent STAT3 activation further reinforces muscle wasting by upregulating caspase-3 and muscle-specific E3 ubiquitin ligases, including MAFbx/Atrogin-1, thereby accelerating ubiquitin–proteasome system-mediated protein degradation [55]. Simultaneously, the β isoform of p38 MAPK activated downstream of TLR4 and inflammatory cytokine signaling phosphorylates p300 (Ser12), leading to activation of the transcription factor C/EBPβ and induction of muscle atrophy-related genes [55]. p38β MAPK also promotes autophagic by phosphorylating ULK1 (Ser555) and upregulates genes involved in lysosomal and proteasomal pathways, such as LC3b and Gabarapl1 [55]. Collectively, these pathways form a feed-forward catabolic signaling network in experimental models of cancer cachexia, in which NF-κB acts as a central coordinating node that integrates inflammatory and stress-responsive inputs rather than functioning as an isolated or exclusive driver. The cooperative activation of NF-κB, STAT3, and p38 MAPK signaling establishes a feed-forward catabolic program that sustains inflammation, proteolysis, and autophagic activation, ultimately driving progressive skeletal muscle wasting in cancer cachexia (Figure 2).

## 4. Pathological Mechanisms of NF-κB in Cancer Cachexia

### 4.1. NF-κB as a Central Regulator of Muscle and Adipose Catabolism in Cancer Cachexia

Cancer cachexia is marked by severe loss of skeletal muscle and adipose tissue, largely driven by persistent systemic inflammation and dysregulated catabolic signaling [56]. Central to this process is aberrant activation of the NF-κB pathway, which integrates inflammatory cues to regulate muscle proteolysis, impaired regeneration, and adipose remodeling [3,54]. In skeletal muscle, elevated levels of TNF-α, IL-1β, and IL-6 activate the IKKβ NF-κB axis, resulting in IκBα degradation and nuclear translocation of p65/p50 heterodimers [57]. These complexes induce expression of catabolic genes such as MuRF1, Atrogin-1, and iNOS, thereby promoting protein degradation through both the UPS and the ALP [6,9,58]. Simultaneously, NF-κB suppresses the IGF-1/Akt/mTOR anabolic axis and inhibits myogenic differentiation via Pax7 upregulation and repression of Cyclin D1 and MyoD [9]. These effects are reinforced by macrophage and fibro-adipogenic progenitor (FAP) infiltration, establishing a feed-forward loop of inflammation and tissue degradation. Muscle proteostasis is maintained by coordinated UPS and ALP activity; however, excessive activation of both pathways regulated in part by FOXO transcription factors exacerbates muscle wasting [59]. The concurrent upregulation of MuRF1 and Atrogin-1 is a hallmark of cachexia and a validated therapeutic target, with their inhibition shown to delay atrophy in preclinical models [60].

In white adipose tissue (WAT), NF-κB contributes to metabolic dysfunction by promoting lipolysis and white-to-brown adipocyte transdifferentiation [61]. Inflammatory cytokines such as TNF-α and IL-6 induce NF-κB activation in adipocytes, upregulating lipolytic enzymes (ATGL, HSL) and thermogenic markers such as UCP1 [62,63]. These changes drive elevated free fatty acid (FFA) release and energy expenditure, further aggravating the negative energy balance characteristic of cachexia [64]. IL-6 further amplifies these effects through the AMPK–HSL and JAK/STAT3 axes, while redox-sensitive mechanisms (e.g., ROS, TLRs) sustain NF-κB activation even in the absence of exogenous stimuli [65,66]. Therapeutically, NF-κB inhibition has shown promise in attenuating both muscle and fat loss [67]. Pharmacologic agents such as DHMEQ, SR12343, and NSAIDs have been reported to suppress inflammation, restore IGF-1/Akt/mTOR signaling, and reduce senescence-associated secretory phenotypes (SASP) in muscles [68]. In adipose tissue, NF-κB blockade via IKKβ inhibition or IκBα overexpression—reduces lipolysis, prevents browning, and preserves adipocyte integrity [69]. Targeting IL-6 signaling provides synergistic benefits by modulating upstream pathways [70,71]. These findings support a multifactorial therapeutic framework targeting the NF-κB centered inflammatory metabolic axis to restore tissue homeostasis, preserve energy stores, and delay cachexia progression [72]. Continued investigation into these strategies may yield novel combinatorial interventions with clinical translational potential [73].

### 4.2. NF-κB as a Central Mediator of Adipose Remodeling and Systemic Metabolic Dysregulation in Cancer Cachexia

Beyond its well-established role in muscle atrophy, NF-κB also orchestrates profound alterations in adipose tissue metabolism and systemic energy homeostasis during cancer cachexia [73]. In white adipose tissue (WAT), pro-inflammatory cytokines particularly TNF-α, IL-1β, and IL-6 activate the canonical IKKβ/NF-κB signaling pathway in adipocytes, leading to the upregulation of lipolytic enzymes such as adipose triglyceride lipase (ATGL) and hormone-sensitive lipase (HSL) [74]. This accelerates triglyceride breakdown and promotes free fatty acid (FFA) release, thereby contributing to elevated energy expenditure and progressive fat loss [75]. Concurrently, NF-κB induces the expression of thermogenic genes like uncoupling protein 1 (UCP1), driving the browning of white adipose tissue and further enhancing futile energy dissipation [63,76]. IL-6 amplifies these effects by simultaneously engaging the AMPK–HSL axis and the JAK/STAT3 pathway, reinforcing both lipolysis and thermogenesis.

Therapeutically, both adipose- and muscle-specific NF-κB inhibition have shown efficacy in reversing cachexia-related metabolic disturbances in preclinical models [77]. Pharmacological agents such as NSAIDs (e.g., indomethacin) and direct NF-κB inhibitors (e.g., DHMEQ, SR12343) have been shown to restore glucose tolerance, improve insulin sensitivity, reduce lipolysis, and preserve adipocyte morphology [78,79,80]. However, given NF-κB’s role in tissue repair and immune defense, systemic inhibition must be carefully calibrated to avoid unwanted side effects, such as impaired regeneration or heightened infection risk [81]. Overall, these findings position NF-κB as a master integrator of inflammatory and metabolic networks in cancer cachexia, as well as a highly actionable target for restoring adipose function and systemic energy homeostasis [82].

### 4.3. NF-κB in Neuro-Metabolic Control of Cachexia

**Figure 3:**
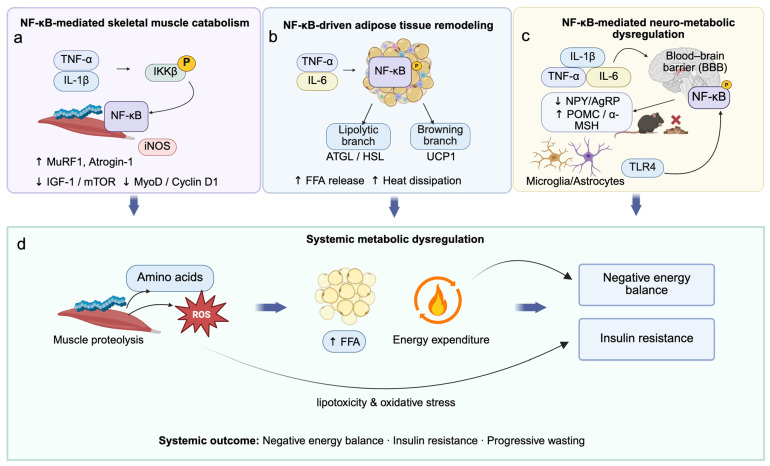
NF-κB–mediated multi-organ metabolic dysregulation in cancer cachexia. Chronic activation of NF-κB related inflammatory signaling is associated with coordinated metabolic dysfunction across multiple organs in cancer cachexia. (**a**) In skeletal muscle, pro-inflammatory cytokines activate NF-κB, inducing MuRF1, Atrogin-1, and *iNOS*, while suppressing IGF-1/mTOR signaling and myogenic regulators, thereby promoting muscle proteolysis and impaired regeneration. (**b**) In adipose tissue, NF-κB drives tissue remodeling through activation of lipolytic pathways (ATGL/HSL) and induction of white adipose tissue browning via UCP1, resulting in increased FFA release and heat dissipation. (**c**) In the central nervous system, cytokine-induced NF-κB activation in hypothalamic neurons and glial cells disrupts neuro-metabolic regulation by suppressing orexigenic (NPY/AgRP) and enhancing anorexigenic (POMC/α-MSH) signaling, contributing to anorexia. (**d**) Collectively, these organ-specific alterations identified predominantly in experimental models of cancer cachexia converge to promote systemic metabolic dysregulation, characterized by elevated energy expenditure, lipotoxicity, oxidative stress, negative energy balance, insulin resistance, and progressive tissue wasting.

Anorexia is a cardinal feature of cancer cachexia that contributes to systemic energy imbalance and worsened clinical outcomes. Central to this process is the activation of hypothalamic NF-κB signaling, which integrates inflammatory and nutritional cues to regulate appetite. Circulating cytokines such as TNF-α, IL-6, and IL-1β cross the blood–brain barrier via active transport or leaky regions like the median eminence and activate NF-κB in hypothalamic neurons [83]. This activation suppresses orexigenic pathways (e.g., ghrelin–NPY/AgRP) and enhances anorexigenic signaling (e.g., POMC/α-MSH), leading to reduced food intake and persistent metabolic suppression. Experimental models have shown that lipopolysaccharide (LPS) or HIV-Tat protein activates NF-κB in POMC neurons, inducing anorexia, while targeted deletion of IKKβ in these neurons alleviates inflammation-induced appetite loss [83]. Beyond neurons, TLR4/NF-κB signaling in hypothalamic microglia and astrocytes drives neuroinflammation, impairs leptin and insulin sensitivity, and disrupts energy homeostasis [83,84]. Endoplasmic reticulum (ER) stress further engages the IKKβ/NF-κB pathway, linking nutrient overload to hypothalamic dysfunction. Given this multifaceted role, NF-κB presents a promising therapeutic target for reversing anorexia in cancer cachexia. Inhibition strategies ranging from natural compounds (e.g., curcumin, EPA) to pharmacologic agents (e.g., NSAIDs) and genetic interventions (e.g., astrocyte-specific IKKβ knockout) have been shown to reduce hypothalamic inflammation, restore orexigenic signaling, and improve food intake in preclinical models [85]. Notably, clinical studies demonstrate that curcumin alleviates anorexia-related symptoms and improves muscle strength in cachectic patients, while agents like CTRP4 restore leptin sensitivity via astrocytic NF-κB modulation [83,86]. Collectively, NF-κB orchestrates muscle catabolism, adipose remodeling, and neuro-metabolic dysregulation, converging on systemic energy imbalance and progressive cachexia (Figure 3).

## 5. NF-κB Inhibition as a Therapeutic Strategy in Cancer Cachexia

NF-κB is a central mediator of inflammation, proteolysis, and metabolic dysfunction in cancer cachexia, making it a highly promising therapeutic target. Several pharmacological agents including small molecule inhibitors, natural compounds, and anti-inflammatory drugs have demonstrated efficacy in preclinical and clinical studies. Key preclinical and clinical studies targeting NF-κB in cancer cachexia are summarized in Table 1. SR12343, a selective IKK/NF-κB inhibitor, significantly attenuated chemotherapy-induced muscle wasting by suppressing NF-κB activation, reducing senescence-associated secretory phenotype (SASP) markers, and preserving lean and fat mass, grip strength, and body weight [87]. Similarly, DHMEQ, which blocks NF-κB p65 DNA-binding activity, alleviated muscle atrophy and systemic inflammation in mouse models. Natural compounds such as curcumin and EPA also exhibit NF-κB inhibitory effects. In breast cancer cachexia models, curcumin downregulated MuRF1 and Atrogin-1, improved mitochondrial function, and preserved muscle fiber integrity [88]. NSAIDs, including indomethacin and ibuprofen, inhibit COX, thereby reducing prostaglandin synthesis and NF-κB-mediated inflammatory signaling. A systematic review indicated that prolonged NSAID use reduced the incidence of cancer cachexia by approximately 23% [89,90]. However, anti–TNF-α therapies (e.g., infliximab, etanercept) have shown inconsistent outcomes, likely due to cytokine redundancy and compensatory activation of IL-6 or IL-1β. In contrast, IL-6 receptor antagonists (e.g., tocilizumab, siltuximab) have demonstrated more consistent benefits, including suppression of systemic inflammation and improvement in muscle function through dual inhibition of NF-κB and STAT3 pathways [91,92,93].

**Figure 4:**
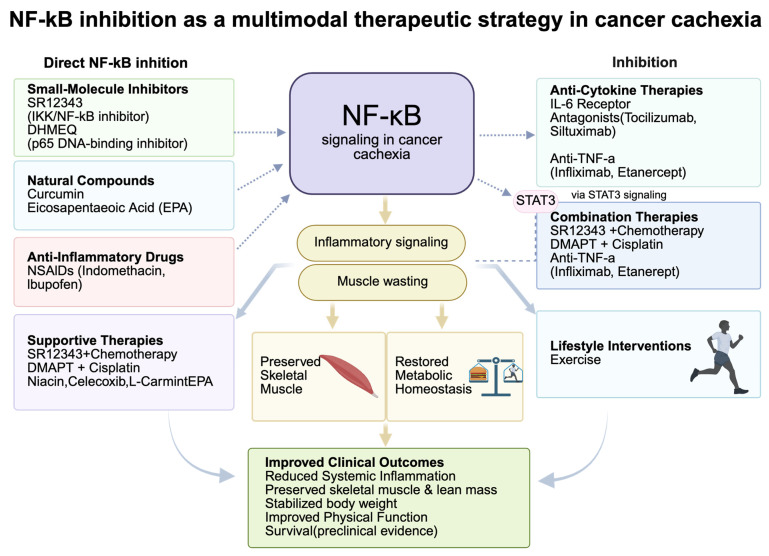
NF-κB inhibition as a multimodal therapeutic strategy in cancer cachexia. NF-κB acts as a central regulator of inflammation, muscle wasting, adipose remodeling, and metabolic dysfunction in cancer cachexia. Therapeutic interventions including small-molecule inhibitors (SR12343, DHMEQ), natural compounds (curcumin, EPA), NSAIDs, anti-cytokine agents, combination regimens, and exercise target NF-κB directly or via upstream pathways (e.g., STAT3). These strategies collectively suppress SASP, reduce systemic inflammation, preserve muscle mass, restore metabolic balance, and improve clinical outcomes.

Given the multifactorial nature of cachexia, combination therapies that concurrently target NF-κB and other pathogenic mechanisms are gaining prominence. Co-administration of SR12343 with chemotherapy reduced muscle atrophy, systemic inflammation, and SASP expression, highlighting its dual role in protecting muscle integrity and enhancing chemotherapy tolerance [59]. Similarly, the combination of DMAPT and cisplatin enhanced chemosensitivity and preserved body weight and renal function in bladder cancer models by reducing IL-6 elevation. Nutritional and anti-inflammatory regimens—such as combinations of niacin, celecoxib, L-carnitine, and EPA have improved lean mass, reduced CRP, TNF-α, and reactive oxygen species, and enhanced patient-reported outcomes [94]. Clinical trials have shown that ibuprofen supplementation can stabilize body mass in patients with lung cancer. Furthermore, dual inhibition of TGF-β and NF-κB has shown efficacy in reducing muscle loss and prolonging survival in pancreatic cancer cachexia models. Exercise, as a non-pharmacologic adjunct, promotes anti-inflammatory myokine expression, reverses systemic inflammation, and mitigates muscle wasting. Collectively, these findings support a multimodal therapeutic paradigm that integrates NF-κB inhibition with chemotherapy, anti-cytokine therapies, nutritional support, and behavioral interventions. This integrative approach holds strong potential to preserve skeletal muscle, restore metabolic homeostasis, and improve clinical outcomes in patients with cancer cachexia (Figure 4).

## 6. Challenges and Future Directions

Despite compelling evidence supporting NF-κB as a central driver of cancer cachexia, accumulating data indicate that its pathological role is highly context-dependent, varying across disease stages, tissue compartments, and cellular subtypes [17,95,96,97]. Here, we propose a unifying NF-κB centered cachectic cascade model, in which temporal and spatial features of NF-κB activation determine its pathological impact across organs. While transient NF-κB activation is essential for immune defense, tissue repair, and adaptive stress responses, sustained low-grade activation characteristic of chronic tumor-associated inflammation drives persistent catabolic signaling, metabolic dysregulation, and progressive tissue wasting [98]. This temporal dichotomy provides a mechanistic framework to reconcile the inconsistent clinical efficacy of systemic anti-inflammatory strategies, particularly anti-TNF-α therapies, despite robust preclinical benefits [4,99,100].

Importantly, NF-κB signaling exerts distinct and sometimes opposing effects depending on the cellular context. Within the proposed cachectic cascade, NF-κB functions as a multicellular signaling hub rather than a uniform effector. In skeletal muscle fibers, chronic activation of the canonical IKKβ–NF-κB pathway directly induces proteolytic gene expression (e.g., *MuRF1*, *Atrogin-1*, *iNOS*) and suppresses anabolic signaling, thereby promoting muscle atrophy [3,13,101]. In contrast, NF-κB activation within infiltrating macrophages, fibro-adipogenic progenitors, and endothelial cells amplifies paracrine cytokine release and extracellular matrix remodeling, indirectly exacerbating muscle degeneration, supporting a multicellular regulation of wasting states [102]. Similarly, hypothalamic NF-κB activation integrates peripheral inflammatory signals to suppress appetite and disrupt neuroendocrine control of energy homeostasis, further accelerating systemic wasting [103]. Together, these observations highlight a spatiotemporally coordinated, multicellular inflammatory network in which NF-κB integrates local and systemic signals to drive cachexia progression [104].

From a translational perspective, this complexity highlights the limitations of indiscriminate NF-κB inhibition. Broad systemic blockade fails to account for disease stage–specific and tissue-specific NF-κB functions and may compromise immune surveillance and regenerative capacity, particularly in patients receiving cytotoxic chemotherapy [105,106]. Consequently, future therapeutic strategies should prioritize spatiotemporal precision, targeting pathological NF-κB activation within specific tissues or disease stages while preserving its physiological functions [107]. Such precision-based approaches represent a conceptual shift from pathway suppression toward context-aware modulation of inflammatory signaling. Approaches such as cell-type-restricted IKKβ modulation, combination therapies targeting upstream cytokine drivers (e.g., IL-6/JAK/STAT3), or integration with metabolic and exercise-based interventions may provide superior efficacy with reduced toxicity [108,109]. Consistent with this notion, the multifactorial nature of cancer cachexia has increasingly shifted therapeutic efforts toward multimodal strategies that simultaneously target inflammatory, metabolic, and oncogenic pathways [110]. Preclinical studies strongly support this paradigm. For instance, co-treatment with the NF-κB inhibitor DMAPT and cisplatin enhanced tumor chemosensitivity while attenuating chemotherapy-induced muscle wasting and systemic IL-6 elevation, preserving both body weight and renal function [111]. Similarly, the IKK/NF-κB inhibitor SR12343, when combined with chemotherapy, significantly reduced muscle atrophy, weight loss, and inflammation by suppressing senescence-associated secretory phenotype (SASP) signaling, highlighting the therapeutic relevance of targeting inflammation-induced senescence under cytotoxic stress [59]. Early-phase clinical interventions have mirrored these findings: regimens combining NSAIDs (e.g., ibuprofen, celecoxib) with metabolic supplements have demonstrated improvements in lean mass and reductions in systemic inflammation [99,112]. Emerging strategies further emphasize synergistic pathway modulation. Dual inhibition of TGF-β and NF-κB signaling in pancreatic cancer cachexia, for example, has demonstrated efficacy in preserving skeletal muscle, mitigating weight loss, and prolonging survival in preclinical models [113,114]. Collectively, these findings reinforce the necessity of addressing tumor burden, chronic inflammation, and metabolic dysregulation in parallel. Moving forward, successful clinical translation will depend on the precise optimization of treatment timing, dosage, and patient stratification to maximize therapeutic benefit while minimizing adverse effects particularly those that may compromise anabolic or regenerative pathways.

Finally, patient stratification based on inflammatory burden may be critical for the success of NF-κB targeted interventions. We propose a clinically actionable stratification framework in which biomarkers such as circulating IL-6, C-reactive protein, or transcriptional NF-κB activity signatures are used to identify patient subsets most likely to benefit from NF-κB modulation. As mechanistic understanding of cachexia deepens, future therapeutic regimens will likely adopt a precision medicine framework, integrating targeted molecular therapies with individualized nutritional support and lifestyle interventions. This approach reframes NF-κB targeted therapy from a palliative measure to a proactive, mechanism-based intervention strategy for cancer cachexia.

## 7. Conclusions

NF-κB functions as a central inflammatory metabolic regulatory hub in cancer cachexia, linking chronic systemic inflammation to skeletal muscle atrophy, impaired regenerative capacity, and global energy imbalance [54]. Pro-inflammatory cytokines such as TNF-α, IL-1β, and IL-6 activate NF-κB-dependent signaling programs that drive transcription of catabolic genes, including *MuRF1*, Atrogin-1, and iNOS, thereby promoting activation of the ubiquitin proteasome system and autophagy lysosome pathway to accelerate muscle protein degradation and fiber atrophy [22,115]. In experimental models of cancer cachexia, sustained activation of the canonical NF-κB pathway disrupts muscle homeostasis not only by enhancing proteolysis but also by impairing regeneration, through increased Pax7 expression and inhibition of MyoD driven myogenesis [9]. Beyond skeletal muscle, NF-κB contributes to systemic metabolic dysfunction by repressing the IGF-1/Akt/mTOR anabolic pathway, promoting hepatic gluconeogenesis, inducing insulin resistance, and enhancing lipolytic enzyme activity [79,116]. It also facilitates white adipose tissue browning through *UCP1* upregulation, further increasing energy expenditure [117]. These events reinforce a feed-forward inflammatory loop, in which NF-κB-driven catabolic and metabolic alterations amplify cytokine production, sustaining chronic low-grade inflammation. Collectively, current evidence derived predominantly from mechanistic and preclinical studies supports a prominent role for NF-κB as a coordinating signaling axis in cancer cachexia, while underscoring the need for further clinical validation to define its causal contribution and therapeutic tractability. Growing evidence supports inhibition of NF-κB related inflammatory signaling as a potentially viable therapeutic strategy for cancer cachexia, particularly when integrated with chemotherapy, anti-inflammatory agents, or metabolic support [118]. The IKK/NF-κB inhibitor SR12343 significantly attenuated chemotherapy-induced muscle wasting, preserved lean mass, and reduced SASP markers in murine models, while similar protective effects were observed with DMAPT cisplatin co-treatment [119]. Importantly, these benefits have been demonstrated predominantly in preclinical settings. In contrast, clinical regimens incorporating nonsteroidal anti-inflammatory drugs (NSAIDs), omega-3 polyunsaturated fatty acids, and metabolic supplements have shown more modest but reproducible benefits, including stabilization of body weight, reduction in systemic inflammatory markers, and improvement in quality-of-life measures in patients with advanced malignancies [120]. Furthermore, targeting convergent inflammatory nodes such as combined inhibition of NF-κB and TGF-β signaling has shown additive benefits in pancreatic cancer cachexia models, including improved survival [114]. Collectively, these findings support integrated, multimodal treatment paradigms targeting NF-κB-centered inflammatory–metabolic networks, while also underscoring the need for rigorously designed, large-scale randomized clinical trials to define optimal timing, dosing, safety profiles, and patient stratification strategies.

## Figures and Tables

**Table 1:** Preclinical and Clinical Studies Targeting NF-κB in Cancer Cachexia.

Study Type	Model/Population	Intervention	NF-κB Target/Mechanism	Cachexia-Related Outcomes	Key Findings	Reference
Preclinical	C26 colon carcinoma mice	Indomethacin (NSAID)	COX inhibition reduced NF-κB activation	Body weight, muscle mass	Reduced muscle wasting and improved body weight	[12]
Preclinical	Chemotherapy-induced cachexia mice	SR12343	IKK/NF-κB inhibition	Lean mass, grip strength, inflammation	Preserved muscle mass and reduced SASP	[59]
Preclinical	Tumor-bearing mice	DHMEQ	NF-κB p65 DNA-binding inhibition	Muscle atrophy, cytokines	Attenuated muscle loss and systemic inflammation	[91]
Clinical	Advanced cancer patients	Ibuprofen	Indirect NF-κB suppression via COX inhibition	Body weight, CRP	Stabilized body mass and reduced inflammation	[90]
Clinical	Cachectic cancer patients	Curcumin	NF-κB/STAT3 inhibition	Appetite, muscle strength	Improved anorexia-related symptoms	[84]
Clinical	Cancer cachexia patients	IL-6R antibodies	Upstream blockade of NF-κB/STAT3 axis	Inflammation, muscle function	Reduced systemic inflammation	[93]

## Data Availability

No new data were created or analyzed in this study.

## References

[1] Blum D., Stene G.B., Solheim T.S., Fayers P., Hjermstad M.J., Baracos V.E., Fearon K., Strasser F., Kaasa S. (2014). Validation of the Consensus-Definition for Cancer Cachexia and evaluation of a classification model—A study based on data from an international multicentre project (EPCRC-CSA). Ann. Oncol..

[2] Yeom E., Yu K. (2022). Understanding the molecular basis of anorexia and tissue wasting in cancer cachexia. Exp. Mol. Med..

[3] Cai D., Frantz J.D., Tawa N.E., Melendez P.A., Oh B.C., Lidov H.G., Hasselgren P.O., Frontera W.R., Lee J., Glass D.J. (2004). IKKbeta/NF-kappaB activation causes severe muscle wasting in mice. Cell.

[4] Webster J.M., Kempen L., Hardy R.S., Langen R.C.J. (2020). Inflammation and Skeletal Muscle Wasting During Cachexia. Front. Physiol..

[5] Langen R.C., Haegens A., Vernooy J.H., Wouters E.F., de Winther M.P., Carlsen H., Steele C., Shoelson S.E., Schols A.M. (2012). NF-κB activation is required for the transition of pulmonary inflammation to muscle atrophy. Am. J. Respir. Cell Mol. Biol..

[6] Knaevelsrud H., Simonsen A. (2010). Fighting disease by selective autophagy of aggregate-prone proteins. FEBS Lett..

[7] Yadav A., Singh A., Phogat J., Dahuja A., Dabur R. (2021). Magnoflorine prevent the skeletal muscle atrophy via Akt/mTOR/FoxO signal pathway and increase slow-MyHC production in streptozotocin-induced diabetic rats. J. Ethnopharmacol..

[8] Zimmers T.A., Fishel M.L., Bonetto A. (2016). STAT3 in the systemic inflammation of cancer cachexia. Semin. Cell Dev. Biol..

[9] He W.A., Berardi E., Cardillo V.M., Acharyya S., Aulino P., Thomas-Ahner J., Wang J., Bloomston M., Muscarella P., Nau P. (2013). NF-κB-mediated Pax7 dysregulation in the muscle microenvironment promotes cancer cachexia. J. Clin. Investig..

[10] Biddinger S.B., Hernandez-Ono A., Rask-Madsen C., Haas J.T., Alemán J.O., Suzuki R., Scapa E.F., Agarwal C., Carey M.C., Stephanopoulos G. (2008). Hepatic insulin resistance is sufficient to produce dyslipidemia and susceptibility to atherosclerosis. Cell Metab..

[11] Cerquone Perpetuini A., Re Cecconi A.D., Chiappa M., Martinelli G.B., Fuoco C., Desiderio G., Castagnoli L., Gargioli C., Piccirillo R., Cesareni G. (2018). Group I Paks support muscle regeneration and counteract cancer-associated muscle atrophy. J. Cachexia Sarcopenia Muscle.

[12] Tisdale M.J. (2009). Mechanisms of cancer cachexia. Physiol. Rev..

[13] Bodine S.C., Baehr L.M. (2014). Skeletal muscle atrophy and the E3 ubiquitin ligases MuRF1 and MAFbx/atrogin-1. Am. J. Physiol. Endocrinol. Metab..

[14] Martin A., Gallot Y.S., Freyssenet D. (2023). Molecular mechanisms of cancer cachexia-related loss of skeletal muscle mass: Data analysis from preclinical and clinical studies. J. Cachexia Sarcopenia Muscle.

[15] Sun S.C. (2017). The non-canonical NF-κB pathway in immunity and inflammation. Nat. Rev. Immunol..

[16] Neshan M., Tsilimigras D.I., Han X., Zhu H., Pawlik T.M. (2024). Molecular Mechanisms of Cachexia: A Review. Cells.

[17] Pryce B.R., Oles A., Talbert E.E., Romeo M.J., Vaena S., Sharma S., Spadafora V., Tolliver L., Mahvi D.A., Morgan K.A. (2024). Muscle inflammation is regulated by NF-κB from multiple cells to control distinct states of wasting in cancer cachexia. Cell Rep..

[18] Setiawan T., Sari I.N., Wijaya Y.T., Julianto N.M., Muhammad J.A., Lee H., Chae J.H., Kwon H.Y. (2023). Cancer cachexia: Molecular mechanisms and treatment strategies. J. Hematol. Oncol..

[19] Li Y., Jin H., Chen Y., Huang T., Mi Y., Zou Z. (2021). Cancer cachexia: Molecular mechanism and pharmacological management. Biochem. J..

[20] Laine A., Iyengar P., Pandita T.K. (2013). The role of inflammatory pathways in cancer-associated cachexia and radiation resistance. Mol. Cancer Res..

[21] Lawrence T. (2009). The nuclear factor NF-kappaB pathway in inflammation. Cold Spring Harb. Perspect. Biol..

[22] Guo Q., Jin Y., Chen X., Ye X., Shen X., Lin M., Zeng C., Zhou T., Zhang J. (2024). NF-κB in biology and targeted therapy: New insights and translational implications. Signal Transduct. Target. Ther..

[23] Sun S.C., Chang J.H., Jin J. (2013). Regulation of nuclear factor-κB in autoimmunity. Trends Immunol..

[24] Sun S.C. (2011). Non-canonical NF-κB signaling pathway. Cell Res..

[25] Ghosh S., May M.J., Kopp E.B. (1998). NF-kappa B and Rel proteins: Evolutionarily conserved mediators of immune responses. Annu. Rev. Immunol..

[26] Beinke S., Ley S.C. (2004). Functions of NF-kappaB1 and NF-kappaB2 in immune cell biology. Biochem. J..

[27] Karin M., Delhase M. (2000). The I kappa B kinase (IKK) and NF-kappa B: Key elements of proinflammatory signalling. Semin. Immunol..

[28] Hayden M.S., Ghosh S. (2008). Shared principles in NF-kappaB signaling. Cell.

[29] Sato S., Sanjo H., Takeda K., Ninomiya-Tsuji J., Yamamoto M., Kawai T., Matsumoto K., Takeuchi O., Akira S. (2005). Essential function for the kinase TAK1 in innate and adaptive immune responses. Nat. Immunol..

[30] Chen Z., Hagler J., Palombella V.J., Melandri F., Scherer D., Ballard D., Maniatis T. (1995). Signal-induced site-specific phosphorylation targets I kappa B alpha to the ubiquitin-proteasome pathway. Genes Dev..

[31] Sakurai H., Chiba H., Miyoshi H., Sugita T., Toriumi W. (1999). IkappaB kinases phosphorylate NF-kappaB p65 subunit on serine 536 in the transactivation domain. J. Biol. Chem..

[32] Xiao G., Harhaj E.W., Sun S.C. (2001). NF-kappaB-inducing kinase regulates the processing of NF-kappaB2 p100. Mol. Cell.

[33] Senftleben U., Cao Y., Xiao G., Greten F.R., Krähn G., Bonizzi G., Chen Y., Hu Y., Fong A., Sun S.C. (2001). Activation by IKKalpha of a second, evolutionary conserved, NF-kappa B signaling pathway. Science.

[34] Herzog S., Hug E., Meixlsperger S., Paik J.H., DePinho R.A., Reth M., Jumaa H. (2008). SLP-65 regulates immunoglobulin light chain gene recombination through the PI(3)K-PKB-Foxo pathway. Nat. Immunol..

[35] Yu H., Lin L., Zhang Z., Zhang H., Hu H. (2020). Targeting NF-κB pathway for the therapy of diseases: Mechanism and clinical study. Signal Transduct. Target. Ther..

[36] Baracos V.E., Martin L., Korc M., Guttridge D.C., Fearon K.C.H. (2018). Cancer-associated cachexia. Nat. Rev. Dis. Primers.

[37] Lee M.K., Choi Y.H., Nam T.J. (2021). Pyropia yezoensis protein protects against TNF-α-induced myotube atrophy in C2C12 myotubes via the NF-κB signaling pathway. Mol. Med. Rep..

[38] Hill J.W., Elias C.F., Fukuda M., Williams K.W., Berglund E.D., Holland W.L., Cho Y.R., Chuang J.C., Xu Y., Choi M. (2010). Direct insulin and leptin action on pro-opiomelanocortin neurons is required for normal glucose homeostasis and fertility. Cell Metab..

[39] Jung Y.J., Isaacs J.S., Lee S., Trepel J., Neckers L. (2003). IL-1beta-mediated up-regulation of HIF-1alpha via an NFkappaB/COX-2 pathway identifies HIF-1 as a critical link between inflammation and oncogenesis. FASEB J..

[40] Cheung W.W., Zheng R., Hao S., Wang Z., Gonzalez A., Zhou P., Hoffman H.M., Mak R.H. (2021). The role of IL-1 in adipose browning and muscle wasting in CKD-associated cachexia. Sci. Rep..

[41] Li Y., Dörmann N., Brinschwitz B., Kny M., Martin E., Bartels K., Li N., Giri P.V., Schwanz S., Boschmann M. (2023). SPSB1-mediated inhibition of TGF-β receptor-II impairs myogenesis in inflammation. J. Cachexia Sarcopenia Muscle.

[42] Ma J.F., Sanchez B.J., Hall D.T., Tremblay A.K., Di Marco S., Gallouzi I.E. (2017). STAT3 promotes IFNγ/TNFα-induced muscle wasting in an NF-κB-dependent and IL-6-independent manner. EMBO Mol. Med..

[43] Kapur S., Marcotte B., Marette A. (1999). Mechanism of adipose tissue iNOS induction in endotoxemia. Am. J. Physiol..

[44] Hoesel B., Schmid J.A. (2013). The complexity of NF-κB signaling in inflammation and cancer. Mol. Cancer.

[45] Baldwin A.S. (1996). The NF-kappa B and I kappa B proteins: New discoveries and insights. Annu. Rev. Immunol..

[46] Karin M., Ben-Neriah Y. (2000). Phosphorylation meets ubiquitination: The control of NF-[kappa]B activity. Annu. Rev. Immunol..

[47] Makarov S.S. (2001). NF-kappa B in rheumatoid arthritis: A pivotal regulator of inflammation, hyperplasia, and tissue destruction. Arthritis Res..

[48] Ghosh S., Hayden M.S. (2008). New regulators of NF-kappaB in inflammation. Nat. Rev. Immunol..

[49] White J.P. (2017). IL-6, cancer and cachexia: Metabolic dysfunction creates the perfect storm. Transl. Cancer Res..

[50] Mao H., Zhao X., Sun S.C. (2025). NF-κB in inflammation and cancer. Cell Mol. Immunol..

[51] Shi H., Kokoeva M.V., Inouye K., Tzameli I., Yin H., Flier J.S. (2006). TLR4 links innate immunity and fatty acid-induced insulin resistance. J. Clin. Investig..

[52] Oeckinghaus A., Ghosh S. (2009). The NF-kappaB family of transcription factors and its regulation. Cold Spring Harb. Perspect. Biol..

[53] Elyaman W., Bassil R., Bradshaw E.M., Orent W., Lahoud Y., Zhu B., Radtke F., Yagita H., Khoury S.J. (2012). Notch receptors and Smad3 signaling cooperate in the induction of interleukin-9-producing T cells. Immunity.

[54] Argilés J.M., Busquets S., Stemmler B., López-Soriano F.J. (2014). Cancer cachexia: Understanding the molecular basis. Nat. Rev. Cancer.

[55] Sin T.K., Zhang G., Zhang Z., Zhu J.Z., Zuo Y., Frost J.A., Li M., Li Y.P. (2021). Cancer-Induced Muscle Wasting Requires p38β MAPK Activation of p300. Cancer Res..

[56] Fearon K., Strasser F., Anker S.D., Bosaeus I., Bruera E., Fainsinger R.L., Jatoi A., Loprinzi C., MacDonald N., Mantovani G. (2011). Definition and classification of cancer cachexia: An international consensus. Lancet Oncol..

[57] Wang H., Chu W.S., Lu T., Hasstedt S.J., Kern P.A., Elbein S.C. (2004). Uncoupling protein-2 polymorphisms in type 2 diabetes, obesity, and insulin secretion. Am. J. Physiol. Endocrinol. Metab..

[58] Bodine S.C., Latres E., Baumhueter S., Lai V.K., Nunez L., Clarke B.A., Poueymirou W.T., Panaro F.J., Na E., Dharmarajan K. (2001). Identification of ubiquitin ligases required for skeletal muscle atrophy. Science.

[59] Englund D.A., Jolliffe A.M., Hanson G.J., Aversa Z., Zhang X., Jiang X., White T.A., Zhang L., Monroe D.G., Robbins P.D. (2024). Senotherapeutic drug treatment ameliorates chemotherapy-induced cachexia. JCI Insight.

[60] Peterson J.M., Bakkar N., Guttridge D.C. (2011). NF-κB signaling in skeletal muscle health and disease. Curr. Top. Dev. Biol..

[61] Argilés J.M., López-Soriano F.J., Stemmler B., Busquets S. (2023). Cancer-associated cachexia-understanding the tumour macroenvironment and microenvironment to improve management. Nat. Rev. Clin. Oncol..

[62] Anderson L.J., Lee J., Anderson B., Lee B., Migula D., Sauer A., Chong N., Liu H., Wu P.C., Dash A. (2022). Whole-body and adipose tissue metabolic phenotype in cancer patients. J. Cachexia Sarcopenia Muscle.

[63] Petruzzelli M., Schweiger M., Schreiber R., Campos-Olivas R., Tsoli M., Allen J., Swarbrick M., Rose-John S., Rincon M., Robertson G. (2014). A switch from white to brown fat increases energy expenditure in cancer-associated cachexia. Cell Metab..

[64] Ahern G.P. (2013). Transient receptor potential channels and energy homeostasis. Trends Endocrinol. Metab..

[65] Brasier A.R. (2010). The nuclear factor-kappaB-interleukin-6 signalling pathway mediating vascular inflammation. Cardiovasc. Res..

[66] Wu Q., Liu Z., Li B., Liu Y.E., Wang P. (2024). Immunoregulation in cancer-associated cachexia. J. Adv. Res..

[67] Karin M., Greten F.R. (2005). NF-kappaB: Linking inflammation and immunity to cancer development and progression. Nat. Rev. Immunol..

[68] Ji Y., Li M., Chang M., Liu R., Qiu J., Wang K., Deng C., Shen Y., Zhu J., Wang W. (2022). Inflammation: Roles in Skeletal Muscle Atrophy. Antioxidants.

[69] Laurencikiene J., van Harmelen V., Nordström E.A., Dicker A., Blomqvist L., Näslund E., Langin D., Arner P., Rydén M. (2007). NF-κB is important for TNF-α-induced lipolysis in human adipocytes. J. Lipid Res..

[70] Zhang Y., Wang J., Wang X., Gao T., Tian H., Zhou D., Zhang L., Li G., Wang X. (2020). The autophagic-lysosomal and ubiquitin proteasome systems are simultaneously activated in the skeletal muscle of gastric cancer patients with cachexia. Am. J. Clin. Nutr..

[71] Sartori R., Romanello V., Sandri M. (2021). Mechanisms of muscle atrophy and hypertrophy: Implications in health and disease. Nat. Commun..

[72] Anderson L.J., Albrecht E.D., Garcia J.M. (2017). Update on Management of Cancer-Related Cachexia. Curr. Oncol. Rep..

[73] Han J., Meng Q., Shen L., Wu G. (2018). Interleukin-6 induces fat loss in cancer cachexia by promoting white adipose tissue lipolysis and browning. Lipids Health Dis..

[74] Ryden M., Dicker A., van Harmelen V., Hauner H., Brunnberg M., Perbeck L., Lonnqvist F., Arner P. (2002). Mapping of early signaling events in tumor necrosis factor-alpha -mediated lipolysis in human fat cells. J. Biol. Chem..

[75] Tsoli M., Robertson G. (2013). Cancer cachexia: Malignant inflammation, tumorkines, and metabolic mayhem. Trends Endocrinol. Metab..

[76] Kir S., White J.P., Kleiner S., Kazak L., Cohen P., Baracos V.E., Spiegelman B.M. (2014). Tumour-derived PTH-related protein triggers adipose tissue browning and cancer cachexia. Nature.

[77] Chen L., Fischle W., Verdin E., Greene W.C. (2001). Duration of nuclear NF-kappaB action regulated by reversible acetylation. Science.

[78] Shoelson S.E., Lee J., Goldfine A.B. (2006). Inflammation and insulin resistance. J. Clin. Investig..

[79] Masi T., Patel B.M. (2021). Altered glucose metabolism and insulin resistance in cancer-induced cachexia: A sweet poison. Pharmacol. Rep..

[80] Sandri M. (2013). Protein breakdown in muscle wasting: Role of autophagy-lysosome and ubiquitin-proteasome. Int. J. Biochem. Cell Biol..

[81] Cawthorn W.P., Sethi J.K. (2008). TNF-alpha and adipocyte biology. FEBS Lett..

[82] Porporato P.E. (2016). Understanding cachexia as a cancer metabolism syndrome. Oncogenesis.

[83] Jang P.G., Namkoong C., Kang G.M., Hur M.W., Kim S.W., Kim G.H., Kang Y., Jeon M.J., Kim E.H., Lee M.S. (2010). NF-kappaB activation in hypothalamic pro-opiomelanocortin neurons is essential in illness- and leptin-induced anorexia. J. Biol. Chem..

[84] Thambamroong T., Seetalarom K., Saichaemchan S., Pumsutas Y., Prasongsook N. (2022). Efficacy of Curcumin on Treating Cancer Anorexia-Cachexia Syndrome in Locally or Advanced Head and Neck Cancer: A Double-Blind, Placebo-Controlled Randomised Phase IIa Trial (CurChexia). J. Nutr. Metab..

[85] Panknin T.M., Howe C.L., Hauer M., Bucchireddigari B., Rossi A.M., Funk J.L. (2023). Curcumin Supplementation and Human Disease: A Scoping Review of Clinical Trials. Int. J. Mol. Sci..

[86] Böttcher M., Müller-Fielitz H., Sundaram S.M., Gallet S., Neve V., Shionoya K., Zager A., Quan N., Liu X., Schmidt-Ullrich R. (2020). NF-κB signaling in tanycytes mediates inflammation-induced anorexia. Mol. Metab..

[87] Zhang X., Zhang G., Zhang H., Karin M., Bai H., Cai D. (2008). Hypothalamic IKKbeta/NF-kappaB and ER stress link overnutrition to energy imbalance and obesity. Cell.

[88] Faiad J., Andrade M.F., de Castro G., de Resende J., Coêlho M., Aquino G., Seelaender M. (2025). Muscle loss in cancer cachexia: What is the basis for nutritional support?. Front. Pharmacol..

[89] Yakovenko A., Cameron M., Trevino J.G. (2018). Molecular therapeutic strategies targeting pancreatic cancer induced cachexia. World J. Gastrointest. Surg..

[90] Olaechea S., Gilmore A., Alvarez C., Gannavarapu B.S., Infante R., Iyengar P. (2022). Associations of Prior Chronic Use of Non-Steroidal Anti-Inflammatory Drugs (NSAIDs) and Glucocorticoids with Cachexia Incidence and Survival. Front. Oncol..

[91] Kuroda K., Horiguchi Y., Nakashima J., Kikuchi E., Kanao K., Miyajima A., Ohigashi T., Umezawa K., Murai M. (2005). Prevention of cancer cachexia by a novel nuclear factor {kappa}B inhibitor in prostate cancer. Clin. Cancer Res..

[92] Kang E.A., Park J.M., Jin W., Tchahc H., Kwon K.A., Hahm K.B. (2022). Amelioration of cancer cachexia with preemptive administration of tumor necrosis factor-α blocker. J. Clin. Biochem. Nutr..

[93] Narsale A.A., Carson J.A. (2014). Role of interleukin-6 in cachexia: Therapeutic implications. Curr. Opin. Support. Palliat. Care.

[94] Huang Z., Zhong L., Zhu J., Xu H., Ma W., Zhang L., Shen Y., Law B.Y., Ding F., Gu X. (2020). Inhibition of IL-6/JAK/STAT3 pathway rescues denervation-induced skeletal muscle atrophy. Ann. Transl. Med..

[95] Iaia N., Noviello C., Muscaritoli M., Costelli P. (2025). Inflammation in cancer cachexia: Still the central tenet or just another player?. Am. J. Physiol. Cell Physiol..

[96] Kadakia K.C., Hamilton-Reeves J.M., Baracos V.E. (2023). Current Therapeutic Targets in Cancer Cachexia: A Pathophysiologic Approach. Am. Soc. Clin. Oncol. Educ. Book.

[97] Liu M., Ren Y., Zhou Z., Yang J., Shi X., Cai Y., Arreola A.X., Luo W., Fung K.M., Xu C. (2024). The crosstalk between macrophages and cancer cells potentiates pancreatic cancer cachexia. Cancer Cell.

[98] Shum A.M., Polly P. (2012). Cancer cachexia: Molecular targets and pathways for diagnosis and drug intervention. Endocr. Metab. Immune Disord. Drug Targets.

[99] Onesti J.K., Guttridge D.C. (2014). Inflammation based regulation of cancer cachexia. Biomed. Res. Int..

[100] Thakir T.M., Wang A.R., Decker-Farrell A.R., Ferrer M., Guin R.N., Kleeman S., Levett L., Zhao X., Janowitz T. (2025). Cancer therapy and cachexia. J. Clin. Investig..

[101] Cornwell E.W., Mirbod A., Wu C.L., Kandarian S.C., Jackman R.W. (2014). C26 cancer-induced muscle wasting is IKKβ-dependent and NF-kappaB-independent. PLoS ONE.

[102] Schiaffino S., Dyar K.A., Ciciliot S., Blaauw B., Sandri M. (2013). Mechanisms regulating skeletal muscle growth and atrophy. FEBS J..

[103] Bodine S.C., Stitt T.N., Gonzalez M., Kline W.O., Stover G.L., Bauerlein R., Zlotchenko E., Scrimgeour A., Lawrence J.C., Glass D.J. (2001). Akt/mTOR pathway is a crucial regulator of skeletal muscle hypertrophy and can prevent muscle atrophy in vivo. Nat. Cell Biol..

[104] Shao A., Wu H., Hong Y., Tu S., Sun X., Wu Q., Zhao Q., Zhang J., Sheng J. (2016). Hydrogen-Rich Saline Attenuated Subarachnoid Hemorrhage-Induced Early Brain Injury in Rats by Suppressing Inflammatory Response: Possible Involvement of NF-κB Pathway and NLRP3 Inflammasome. Mol. Neurobiol..

[105] Clamon G., Byrne M.M., Talbert E.E. (2022). Inflammation as a Therapeutic Target in Cancer Cachexia. Cancers.

[106] Patel H.J., Patel B.M. (2017). TNF-α and cancer cachexia: Molecular insights and clinical implications. Life Sci..

[107] Zhang L., Bonomi P.D. (2024). Immune System Disorder and Cancer-Associated Cachexia. Cancers.

[108] Jin Y., Lu L., Hua H., Chen B., Fang W., Lin K., Ren P., Geng Z., Wang L., Yan X. (2025). Qingxie Fuzheng granules attenuate cancer cachexia by restoring gut microbiota homeostasis and suppressing IL-6/NF-κB signaling in colorectal adenocarcinoma. Hereditas.

[109] Straughn A.R., Kakar S.S. (2019). Withaferin A ameliorates ovarian cancer-induced cachexia and proinflammatory signaling. J. Ovarian Res..

[110] Fearon K.C. (2008). Cancer cachexia: Developing multimodal therapy for a multidimensional problem. Eur. J. Cancer.

[111] Skipworth R.J., Stewart G.D., Dejong C.H., Preston T., Fearon K.C. (2007). Pathophysiology of cancer cachexia: Much more than host-tumour interaction?. Clin. Nutr..

[112] Arends J., Strasser F., Gonella S., Solheim T.S., Madeddu C., Ravasco P., Buonaccorso L., de van der Schueren M.A.E., Baldwin C., Chasen M. (2021). Cancer cachexia in adult patients: ESMO Clinical Practice Guidelines (☆). ESMO Open.

[113] Del Fabbro E. (2019). Combination therapy in cachexia. Ann. Palliat. Med..

[114] Greco S.H., Tomkötter L., Vahle A.K., Rokosh R., Avanzi A., Mahmood S.K., Deutsch M., Alothman S., Alqunaibit D., Ochi A. (2015). TGF-β Blockade Reduces Mortality and Metabolic Changes in a Validated Murine Model of Pancreatic Cancer Cachexia. PLoS ONE.

[115] Rao V.K., Das D., Taneja R. (2022). Cancer Cachexia: Signaling and Transcriptional Regulation of Muscle Catabolic Genes. Cancers.

[116] Hotamisligil G.S. (2006). Inflammation and metabolic disorders. Nature.

[117] Henriques F., Lopes M.A., Franco F.O., Knobl P., Santos K.B., Bueno L.L., Correa V.A., Bedard A.H., Guilherme A., Birbrair A. (2018). Toll-Like Receptor-4 Disruption Suppresses Adipose Tissue Remodeling and Increases Survival in Cancer Cachexia Syndrome. Sci. Rep..

[118] Park H.S., Kang B., Chon H.J., Im H.S., Lee C.K., Kim I., Kang M.J., Hwang J.E., Bae W.K., Cheon J. (2021). Liposomal irinotecan plus fluorouracil/leucovorin versus FOLFIRINOX as the second-line chemotherapy for patients with metastatic pancreatic cancer: A multicenter retrospective study of the Korean Cancer Study Group (KCSG). ESMO Open.

[119] Gil da Costa R.M., Levesque C., Bianchi-Frias D., Chatterjee P., Lam H.M., Santos C., Coleman I.M., Ferreirinha P., Vilanova M., Pinto da Cunha N. (2023). Pharmacological NF-κB inhibition decreases cisplatin chemoresistance in muscle-invasive bladder cancer and reduces cisplatin-induced toxicities. Mol. Oncol..

[120] Madeddu C., Dessì M., Panzone F., Serpe R., Antoni G., Cau M.C., Montaldo L., Mela Q., Mura M., Astara G. (2012). Randomized phase III clinical trial of a combined treatment with carnitine + celecoxib ± megestrol acetate for patients with cancer-related anorexia/cachexia syndrome. Clin. Nutr..

